# Implementing a Healthy Food Distribution Program: A Supply Chain Strategy to Increase Fruit and Vegetable Access in Underserved Areas

**DOI:** 10.5888/pcd15.170291

**Published:** 2018-05-24

**Authors:** Amelia R. DeFosset, Allison Kwan, Daniel Rizik-Baer, Luis Gutierrez, Lauren N. Gase, Tony Kuo

**Affiliations:** 1 Los Angeles County Department of Public Health, Division of Chronic Disease and Injury Prevention, Los Angeles, California; 2 Leadership for Urban Renewal Network, Los Angeles, California; 3 Spark Policy Institute, Denver, Colorado; 4 University of California Los Angeles, Jonathan and Karin Fielding School of Public Health, Department of Epidemiology, Los Angeles, California; 5 University of California Los Angeles, David Geffen School of Medicine, Department of Family Medicine, Los Angeles, California

## Abstract

Increasing access to fresh produce in small retail venues could improve the diet of people in underserved communities. However, small retailers face barriers to stocking fresh produce. In 2014, an innovative distribution program, Community Markets Purchasing Real and Affordable Foods (COMPRA), was launched in Los Angeles with the aim of making it more convenient and profitable for small retailers to stock fresh produce. Our case study describes the key processes and lessons learned in the first 2 years of implementing COMPRA. Considerable investments in staff capacity and infrastructure were needed to launch COMPRA. Early successes included significant week-to-week increases in the volume of produce distributed. Leveraging partnerships, maintaining a flexible operational and funding structure, and broadly addressing store owners’ needs contributed to initial gains. We describe key challenges and next steps to scaling the program. Lessons learned from implementing COMPRA could inform other jurisdictions considering supply-side approaches to increase access to healthy food.

## Access to Healthy Food in Small Retail Stores

Although a diet high in fruits and vegetables is a key component of a healthy lifestyle, most Americans do not meet dietary recommendations for consumption of fresh produce. The lowest consumption rates are in low-income communities (1). One factor that may contribute to this disparity in low-income communities is the limited availability of full-service grocery stores and the higher concentration of small grocers (eg, corner stores), who may not sell fresh fruits and vegetables or may stock produce that is more expensive or of lower quality relative to full-service grocers (2). Research suggests that small retailers face multiple barriers to stocking fresh produce, including perceived lack of customer demand, limited expertise and infrastructure (eg, refrigeration, signage) to properly store or effectively merchandise fresh produce, and limited purchasing power to access competitive wholesale prices available to full-size grocers (3,4).

Augmenting food distribution systems to work more favorably for small retailers may reduce obstacles they face in stocking fresh produce, especially when paired with complementary strategies that build store owners’ capacity to operate a successful produce business. Although several communities have tested and established these types of healthy food distribution programs (3,4), the elements needed to successfully launch and grow a program have not been fully described in the literature. We conducted a case study to address this gap by describing the key processes and lessons learned from the development and implementation of a healthy food distribution program in Los Angeles during its first 2 years of operation. Potential programmatic next steps and strategies to achieve long-term sustainability are discussed.

## Key Elements: What Does a Healthy Food Distribution Program Provide?

Community Markets Purchasing Real and Affordable Foods (COMPRA) is a healthy food distribution program that was started as a joint venture of the Los Angeles Food Policy Council (LAFPC), Leadership for Urban Renewal Network (LURN) and Asian Pacific Islander Forward Movement (APIFM). COMPRA provides 2 key benefits to participating retailers: distribution services and technical assistance. COMPRA provides these services in parallel to affect a number of primary program outputs and expected outcomes for both store owners and community members (Figure).

**Figure:**
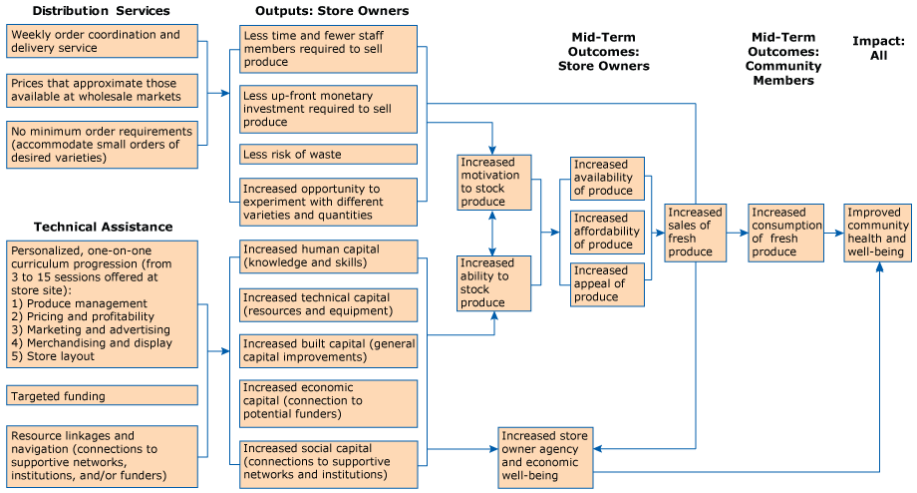
Key program activities, potential outcomes, and effects of the COMPRA healthy food distribution program.

First, COMPRA provides distribution services designed to meet the needs of small retailers — a sector that was previously left out of the produce distribution market in Los Angeles. Distribution services are intended to remove the logistical barriers store owners face to offering fresh produce, by coordinating all aspects of weekly ordering and product delivery and by making every effort possible to secure products at wholesale prices without minimum order requirements (accommodating small orders of desired varieties). These services may reduce required staff time, up-front monetary investment, and the risk of waste associated with stocking fresh produce, providing an opportunity for store owners to experiment with different varieties and quantities of fruits and vegetables until they find the right product mix for their customers.

Second, COMPRA provides a suite of technical assistance services, including one-on-one training using a structured curriculum progression, targeted funding, and resource linkage and navigation. This comprehensive approach, tailored to each store owner’s needs, time, and the demands of running her or his business, is intended to build capacity in 5 areas: 1) human capital (knowledge and skills around selling fresh produce), 2) technical capital (resources and equipment needed to sell fresh produce such as refrigeration, produce baskets, shelving, and signage), 3) built capital (general capital improvements to support business operations), 4) economic capital (connection to financing institutions), and 5) social capital (connections to supportive networks and institutions including neighborhood agencies and city departments). By following a community empowerment model (5), COMPRA expects that developing store owners’ capabilities as community health leaders and business owners may increase their ability to stock produce and improve their personal agency and economic well-being.

Together, distribution and technical assistance services may simultaneously increase store owners’ motivation and ability to stock fresh produce, leading to increases in the availability, affordability, and appeal of produce in their stores. Subsequent increases in sales of these items could ultimately lead to increased consumption by community members and improved community health and well-being.

## The Process: Establishing a Healthy Food Distribution Program

In early 2014, LAFPC received a small amount of unrestricted seed funding as part of its ongoing work to increase healthy food access and equity. This funding came at a time of growing recognition locally of the supply chain barriers small retailers face in sourcing fresh produce. LAFPC contracted with LURN to develop a project designed to reduce these barriers and recruited APIFM to provide additional support, thus forming the operating team. As a first step, the operating team sought to understand the unique issues around sourcing fresh produce in the region. The team conducted focus groups with local agencies and store owners participating in the Healthy Neighborhood Market Network (HNMN), a learning community convened by LAFPC to build the capacity of independently owned small retailers to offer healthier produce options. These discussions identified a pressing need for a distribution system that could provide a convenient and profitable way for small retailers to source fresh produce.

COMPRA was launched as a pilot in late 2014, delivering produce to 7 stores. Each organization on the operating team contributed unique resources that were critical to getting the program off the ground; for instance, APIFM provided a delivery van and LAFPC leveraged existing partnerships with retailers through HNMN to begin recruitment. Working through existing partner networks, the team secured a large supplier willing to provide products at cost and accommodate small-volume orders. The 7 members of the initial group were recruited by using a combined snowball and convenience sampling approach in which participants were identified through existing networks (eg, HNMN) and asked to provide referrals to other potentially interested retailers. Personnel resources, contributed on an in-kind basis by LAFPC and LURN, represented the other primary up-front investment. Significant staff time and expertise were needed to outreach to small retailers; process orders; pick up, sort, and deliver produce; and provide technical assistance to member stores (approximately 2 full-time–equivalent staff members were needed to launch the program). To refine and scale up the program following the pilot phase, it became clear that additional staffing (eg, paid delivery drivers) and equipment (eg, cold storage, delivery vans) were needed. To acquire these resources and to fully support operations, COMPRA secured a diverse combination of funding streams by summer 2015, blending local and national government and foundation resources. With this additional operational capacity, recruitment expanded beyond the original group of members. Stores were identified (by using the same approach as in the pilot phase) and recruited in designated “Promise Zones” in Los Angeles (Hollywood, East Hollywood, Koreatown, Westlake, and Pico Union) — high-poverty, predominantly racial/ethnic and linguistic minority neighborhoods with a high burden of chronic disease (6,7). Table 1 provides additional details of the program development and implementation process.

**Table 1:** Key Activities and Timeline for Establishing the COMPRA Healthy Food Distribution Program in Los Angeles, California, 2014–2016

Project Phase	Key Activities	Approximate Number of Months Required
1. Forming the operating team, designing the concept, and planning for launch	• Received unrestricted seed funding to address food access issues	6
	• Identified interest among stakeholder network in developing a program to address supply chain challenges related to healthy food	
	• Secured LURN as partner to co-lead and prototype the program	
	• Conducted formative research (eg, focus groups) to capture the perspective of stakeholders, including potential program participants	
	• Defined the program scope: focused on establishing a healthy food distribution program	
	• Conducted subsequent research to understand structure of healthy distribution programs in other jurisdictions and issues related to sourcing fresh produce in Los Angeles	

2. Program pilot launch	• Finalized leadership structure: LAFPC, LURN, and APIFM	6–8
	• Secured sufficient resources to begin operations: one delivery van; basic sales-tracking measures; temporary cold storage; staff time to conduct outreach to small retailers, process orders, deliver produce, and provide technical assistance to member stores once they enrolled in the program	
	• Recruited sample of 7 members, including several “member champions,” through Healthy Neighborhood Market Network	
	• Identified produce supplier willing to accommodate small volume orders and provide products at cost	
	• Began accepting and delivering orders to stores	

3. Refining operations and scaling up	• Secured needed resources to sustain and grow operations: 1 additional delivery van, paid delivery drivers, permanent cold storage facilities	≥12
	• Secured local and national funding that supported program operations from US Department of Agriculture, Centers for Disease Control and Prevention (via the Los Angeles County Department of Public Health), The Ahmanson Foundation, and a Community Development Block Grant from City of Los Angeles	
	• Outreached to and recruited additional member stores in Promise Zone areas of Los Angeles	

Abbreviations: APIFM, Asian Pacific Islander Forward Movement; COMPRA, Community Markets Purchasing Real and Affordable Foods; LAFPC, Los Angeles Food Policy Council; LURN, Leadership for Urban Renewal Network.

## Assessing Progress: Early Successes and Evaluation Results

COMPRA’s first 2 years of operation were characterized by steady growth and progress. The program experienced several key operational successes during this time, including standardizing order-taking and management processes, adjusting technical assistance services to better meet store owners’ needs, and developing safety and standard operating procedures. Membership in COMPRA increased in parallel with the evolving operations; by January 2017 the program was delivering to 27 active members (up from 7 when pilot deliveries began in late 2014).

Preliminary program evaluation, conducted in partnership with the Los Angeles County Department of Public Health (LACDPH), used sales data to examine trends in the volume of produce distributed by COMPRA over a one-year period (August 2015–August 2016). In addition, the evaluation team collected comparison price data, by abstraction of publicly available wholesale market prices and local grocery store audits, to understand how prices available through COMPRA compared with those of store owners’ previous vendors. Results (available elsewhere [8]) demonstrated that prices offered to members approximated those at wholesale markets and were lower than prices at full-service grocers. Despite variation at the store level and product level, the total volume of produce distributed by COMPRA increased by an estimated 6 pounds per week over the study period; this change was significant (95% confidence interval, 4.50–7.50) (8) (Table 2).

**Table 2:** Quarterly Changes in Membership and Sales Volume During COMPRA Foods Evaluation Period, Los Angeles, California, 2015–2016^a^

Measure	Quarter				Week-to-Week Change: Parameter Estimate (95% Confidence Interval)^b^
	1	2	3	4	
Average weekly sales volume, lb (SD)	151.92 (81.78)	269.87 (67.90)	280.87 (71.61)	400.25 (111.63)	6.00 (4.50–7.50)^c^
Average no. members per week (range)	10.00 (8–12)	10.38 (10–11)	11.00 (10–12)	13.85 (12–15)	0.10 (0.08–0.12)^c^

Abbreviation: COMPRA, Community Markets Purchasing Real and Affordable Foods.

a Adapted with permission of Springer from DeFosset et al (8).

b Simple linear regression model.

c Significant at α = 0.05.

## Reflections: Challenges, Lessons Learned, and Strategies for Growth and Sustainability

Early program successes demonstrate the potential for public health practitioners to leverage social enterprise models to create changes in the availability of fresh produce in small retail settings. Key challenges in implementing COMPRA included lack of institutional knowledge of the produce distribution sector, the complex nature of the produce business, the need for large up-front investments in equipment and infrastructure, difficulty obtaining the right type of funding, working with small retail store owners, and low consumer demand for fresh produce (Appendix). COMPRA's focus on leveraging existing partnerships, maintaining a flexible organizational culture and funding model, and building store owner trust by addressing their needs broadly helped the program make progress despite these identified barriers.

 Moving forward, COMPRA administrators are committed to making the program a profitable, self-sustaining social enterprise while still providing participating stores with high-quality products at affordable prices. COMPRA has identified, and is currently testing, 5 priority strategies to help achieve this goal. Where feasible, administrators have developed targets for each strategy through a forecasting process that takes into account profit margins, projected growth in membership and sales, and food industry trends:

1. **Connect directly with farmers or growers.** Despite establishing a strong partnership with a large national produce distributor, COMPRA is exploring the possibility of sourcing directly from a network of local farmers and producers to lower costs while maintaining quality. Sourcing from an estimated 5 to 10 farmers could provide the optimal balance between any potential cost savings associated with a shorter distribution chain and the added complexity of sourcing from multiple, small-scale producers. Alternatively, partnering with a distributor of local products, a growers’ association, or a comparable agency could also provide an efficient mechanism to tap into local markets.
2. **Streamline labor.** As the program matures, the COMPRA staff is designing and implementing better management systems to reduce the amount of labor required to operate the program. For example, COMPRA has implemented a custom ordering system that aims to reduce the time needed to take and track orders by 20%. In addition, administrators are developing new and potentially more efficient technical assistance elements, such as small group trainings hosted at participating stores and training delivery drivers to provide preliminary technical assistance. Greater operational efficiency could allow administrators to spend less time on day-to-day logistics and to invest capacity instead in growth activities, including business development and strategic member recruitment. Simultaneously, administrators have established tracking systems to more systematically examine the impact of program changes (eg, new technical assistance processes) on operations costs and program outcomes (eg, store ordering).
3. **Diversify product offerings.** To help increase overall profit margins, COMPRA will begin to distribute higher-margin, more shelf-stable products, including healthy packaged snacks and prepared foods. COMPRA has begun developing this new product line through real-time testing and close consultation with store owners to determine a product mix that is both profitable and appealing to community members. While the exact product catalog is being refined, it is estimated that shifting toward 30% non-produce items could provide a sufficient cushion to account for the less stable profits associated with produce sales. Because determining which packaged and prepared foods count as “healthy” is more complex than with fresh produce, COMPRA is working closely with subject-matter experts at LACDPH to define nutrition standards for their product lines. A key next step will be to develop materials that help communicate technical nutrition criteria to participating store owners (eg, explaining why one granola bar may count as healthy, and another does not) and identifying vendors to provide a consistent supply of products matching these criteria.
4. **Target high-volume stores:** To generate more revenue and increase sales efficiency, COMPRA is moving to a more strategic recruitment model, with the goal of having at least 50% of the member base ordering in the weekly $45 to $50 range. Engaging stores that order at high volumes but require minimal technical assistance can further optimize revenue. Meeting or exceeding these recruitment goals could subsidize the costs of providing services to less-revenue–generating members, keeping them engaged, so as to gradually increase their motivation and ability to offer fresh produce.
5. **Invest in marketing:** Developing a professional, long-term marketing structure to support operations at participating stores could help address low consumer demand for healthy products. This type of marketing effort will likely require significant and regular investment. COMPRA is in the early, exploratory phases of identifying, in consultation with business experts, a marketing model most likely to be both effective and sustainable. However, broad and complementary systems and policy changes are likely needed to significantly increase community demand for and consumption of fresh produce (Table 2).

The experience of COMPRA demonstrates that a healthy food distribution program can reduce the barriers that many small retailers face to stocking fresh produce, potentially increasing their motivation and ability to make healthy options available in underserved communities. COMPRA’s program strengths were identified as the ability to leverage existing partnerships, maintain a flexible organizational culture and funding model, and focus on building trust and addressing the most pressing needs of store owners. Complementary efforts may be needed to optimize the benefits of healthy food distribution programs for community health. Examining the programmatic and operational benchmarks needed to affect consumer behavior (eg, purchase and consumption of healthy foods) and achieve sustainability is a key next step to advance healthy food distribution programs locally. As national interest grows in developing similar supply chain approaches to improve access to healthy foods, the lessons learned from COMPRA’s implementation in Los Angeles provide a practice-based example that can inform planning in other jurisdictions.

## Appendix Key Challenges and Lessons Learned in the Implementation of the Community Markets Purchasing Real and Affordable Foods Program, Los Angeles, California, 2014–2016

**Challenge:**

**Lack of institutional knowledge of produce distribution sector**

Interfacing with produce distribution companies is different from buying food from a supermarket — it is critical to be well-versed in complex sector-specific policies and procedures (eg, account or licensing requirements)

**Lessons Learned:**

**There is likely to be a steep learning curve for nonprofit- or government-based program administrators as they begin to navigate the produce distribution sector**

• Being able to pilot and prototype the COMPRA model before full launch allowed administrators to partially address this challenge

• An adaptable organizational culture and the availability of initial, unrestricted seed funding facilitated the pilot/prototype process

**Challenge:**

**Complex nature of produce business**

• Price and quality instability

• Existing infrastructure and systems within the produce sector are not designed to operate optimally at small scales (eg, cold storage facilities are only available for high-volume clients)

• Delicate/perishable nature of produce

• Special equipment needs

• High risk of waste

**Lessons Learned:**

**Navigating the volatile produce market requires the ability to remain highly flexible in the planning and execution of all aspects of program operations**

• Scaling up the program (eg, purchasing and distributing at higher volumes) may address some operational challenges by making it easier to access special equipment and facilities

• Profitability is likely to remain a challenge, requiring program adjustments to increase revenue and decrease costs

• Short-term adjustments include rethinking the types of boxes used for deliveries, shifting between distributors for different products, and making adjustments to the delivery schedules with minimal notice

• Long-term adjustments include diversifying product lines to include more stable, higher-margin products and streamlining operations

• As a social enterprise, COMPRA faces the challenge of achieving profitability while staying true to its mission to provide high quality, healthy products, at the most affordable prices possible

**Challenge:**

**Need for large up-front investments in equipment and infrastructure to handle produce (eg, delivery vans, cold storage, paid drivers)**

Obtaining capital is difficult as both an untested small business and as a nonprofit

**Lessons Learned:**

**Leveraging partner relationships and available grant and seed funding is crucial to obtain the minimal equipment and resources needed to launch a healthy food distribution program**

• COMPRA benefitted from its position within an established and extensive network of existing local partnerships when launching the program

• Obtaining additional grant funding to secure needed infrastructure and resources was key to expand and formalize the program beyond the initial launch phase

**Challenge:**

**Difficultly obtaining the right funding**

• Funding streams typically available for nonprofit health initiatives are restrictive or not well matched for COMPRA’s model

• Funding from federal health agencies largely cannot be allocated to purchase equipment or fund capital improvements, components that have been key to getting store owners on board

**Lessons Learned:**

**Blending more than one type and source of funding may allow some flexibility in how funding is allocated**

• COMPRA currently operates under a mix of foundation and grant funding

• Managing multiple funding streams is a time- and resource-intensive task

• Moving forward, limiting dependence on grant funding is a priority

**Challenge:**

**Difficulty working with small retail store owners**

• Little financial cushion (not oriented to take risks or experiment)

• Minimal time, staff, or other resources (need lots of support to implement program elements)

• Language barriers

• Previous negative experiences with government/community development programs

**Lessons Learned:**

**Small retail store owners may be willing to participate in new initiatives, provided programs can build trust and demonstrate that they add value from a business standpoint**

• Building trust and demonstrating value may involve providing:

• Access to capital improvement funding and economic resources (beyond those related to produce vending)

• On-site technical assistance tailored around store owners’ needs and schedules

• Linguistically appropriate services (COMPRA has capacity in Spanish and Korean)

• Consistent delivery of desired produce items (eg, culturally appropriate varieties that are appealing to existing customers)

• Although critical, administering these program components are time- and resource-intensive

**Challenge:**

**Low consumer demand for fresh produce**

• High competition from unhealthy products

• Even if available, community members may not have the motivation or ability to select and consume fresh produce

**Lessons Learned:**

**Substantial local, state, and federal policy/systems changes and investments are needed to increase consumer demand for healthy options and reduce competition from unhealthy options **

At a minimum, complementary efforts may be needed to address issues around:

• Consumer education

• Marketing of healthy products as compared with unhealthy products

• Pricing and agricultural policy
